# Associations between aggregate index of systemic inflammation and endometriosis risk utilizing logistic regression analysis

**DOI:** 10.3389/fmed.2026.1817928

**Published:** 2026-05-04

**Authors:** Jing Chen, Kaiqiang Xu, Yanyan Xing, Songping Liu, Lijuan Lu

**Affiliations:** 1 Department of Gynecology and Obstetrics, Jinshan Hospital of Fudan University, Shanghai, China; 2 Qinhuangdao Center for Disease Control and Prevention, Qinhuangdao, Hebei, China

**Keywords:** aggregate index of systemic inflammation, endometriosis, inflammatory biomarker, NHANES, prediction model

## Abstract

**Objective:**

Systemic inflammatory response plays a key role in the pathogenesis of endometriosis. This study aimed to investigate the association between the aggregate index of systemic inflammation (AISI), defined as neutrophil count × platelet count × monocyte count/lymphocyte count, and the risk of endometriosis, and to develop a predictive nomogram incorporating AISI.

**Methods:**

A retrospective analysis was conducted on patients presenting with symptoms suggestive of endometriosis at Jinshan Hospital of Fudan University from January 2023 to December 2025. Participants were classified into endometriosis and non-endometriosis groups based on pathological diagnosis. Baseline characteristics, lipid profiles, and complete blood cell counts were collected. Univariate logistic regression was first performed, followed by multivariate logistic regression with a backward stepwise approach. Model performance was assessed using the area under the receiver operating characteristic curve (AUC), calibration curves, and decision curve analysis. External validation was conducted using data from the National Health and Nutrition Examination Survey (NHANES, 2001–2006).

**Results:**

A total of 226 patients were included, with 104 (46.02%) diagnosed with endometriosis. Compared to the non-endometriosis group, patients with endometriosis had significantly higher age, total cholesterol (TC), total triglycerides (TG), low-density lipoprotein cholesterol (LDL) and AISI levels, and significantly lower body mass index (BMI), high-density lipoprotein cholesterol (HDL), red blood cell count (RBC) and earlier age at menarche (all *p* < 0.05). Multivariate analysis identified age (OR: 1.08, 95%CI: 1.02–1.14, *p* = 0.005), BMI (OR: 0.74, 95%CI: 0.64–0.85, *p* < 0.001), age at menarche (OR: 0.58, 95%CI: 0.43–0.79, *p* < 0.001), RBC (OR: 0.19, 95%CI: 0.06–0.54, *p* = 0.003), log2-AISI (OR: 4.18, 95%CI: 2.42–7.22, *p* < 0.001), TC (OR: 2.31, 95%CI: 1.40–3.82, *p* = 0.001) and HDL (OR: 0.10, 95%CI: 0.03–0.32, *p* < 0.001) as independent predictors of endometriosis risk. The nomogram showed good calibration and strong discriminatory ability, with an AUC of 0.869 (95% CI: 0.823–0.915), which outperformed models excluding AISI (AUC 0.811) or using AISI alone (AUC 0.721). Decision curve analysis demonstrated high clinical utility of the nomogram. External validation from NHANES confirmed that log2-AISI remained independently associated with endometriosis (OR: 2.38, 95% CI: 1.90–2.98, *p* < 0.001) after full adjustment.

**Conclusion:**

AISI is significantly associated with the risk of endometriosis, and the nomogram incorporating AISI with clinical and lipid parameters provides an objective tool for early identification of high-risk individuals.

## 1 Introduction

Endometriosis is a chronic, estrogen-dependent inflammatory disease characterized by the presence of endometrial-like tissue (glands and stroma) outside the uterine cavity, primarily manifesting as persistent pelvic pain, dysmenorrhea, ovulatory dysfunction, and even infertility (1–3). According to statistics, endometriosis affects approximately 10% of women of reproductive age (4). Notably, a trend toward diagnosis at younger ages has been observed in recent years (5). Studies indicate that approximately 10% of female adolescents aged 12–20 exhibit ultrasound features suggestive of endometriosis (6), with the detection rate rising to approximately 40% among those reporting dysmenorrhea (7). This condition is strongly associated with infertility, affecting approximately 40% of women with endometriosis (8), while also leading to reduced quality of life, decreased work productivity, and increased healthcare utilization among affected patients (9, 10). Furthermore, the current average diagnostic delay for endometriosis remains as long as 4–12 years (11). Therefore, early identification of endometriosis plays a crucial role in delaying disease progression, alleviating symptoms, and reducing the overall disease burden.

The pathogenesis of endometriosis involves multifactorial interactions, including genetic predisposition, hormonal dysregulation, and environmental factors (12, 13). Among these, inflammation is recognized as a hallmark feature of endometriosis, with pro-inflammatory cytokines playing a key role in lesion establishment, maintenance, and pain generation (14, 15). Recent studies have indicated that various factors associated with the risk of endometriosis act through inflammatory pathways, such as endocrine-disrupting chemical exposure, lipid metabolism disturbances, and immune dysregulation (16, 17). The aggregate index of systemic inflammation (AISI) has been introduced as a novel composite inflammatory biomarker, calculated using the formula AISI = (neutrophil count × platelet count × monocyte count)/lymphocyte count. This index reflects the comprehensive equilibrium between systemic inflammatory activation and immune status (18, 19). Accumulating evidence has validated AISI as a significant prognostic indicator across diverse pathological conditions, including cardiovascular diseases, idiopathic pulmonary fibrosis, and infectious disorders, where it demonstrates robust associations with disease severity and clinical outcomes (20–22). However, the relationship between AISI and the risk of endometriosis remains unclear. Therefore, this study aims to investigate the association between AISI and the risk of endometriosis, providing an evidence-based foundation for its potential utility as a supplementary biomarker in the early identification of this debilitating condition.

## 2 Methods

### 2.1 Study population

**Figure 1:**
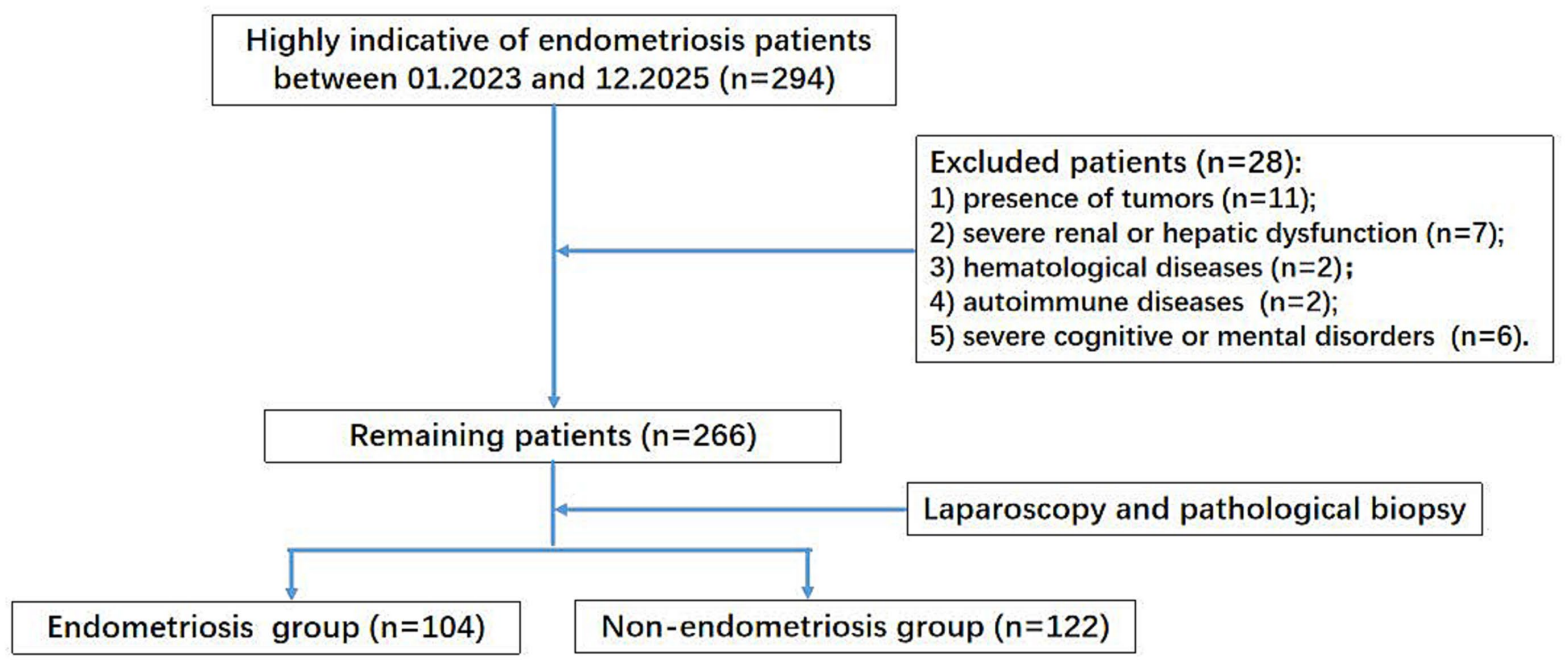
Research flowchart of the study.

This study was a retrospective cohort study that enrolled 294 female patients who presented at Jinshan Hospital of Fudan University between January 2023 and December 2025 due to symptoms suggestive of endometriosis. The inclusion criteria were: (1) age ≥ 18 years; (2) presence of clinical manifestations highly indicative of endometriosis, including chronic pelvic pain, dysmenorrhea, and a pelvic mass detected via physical examination or imaging; (3) underwent laparoscopic evaluation and the diagnosis was confirmed by histological examination; (4) non-smoking and non-drinking status; (5) non-hypertension, non-diabetes or non-coronary heart disease; (6) complete clinical data. The exclusion criteria were: (1) presence of tumors; (2) severe renal or hepatic dysfunction; (3) hematological diseases; (4) autoimmune diseases; (5) severe cognitive or mental disorders. This study was approved by the Institutional Ethics Committee (Approval No. JIEC 2025-S92) and all participants provided written consent. The study flowchart was shown in Figure 1.

### 2.2 Data collection

Data were extracted from the Electronic Medical Record, encompassing baseline characteristics (e.g., age), lifestyle factors (e.g., smoking, alcohol use), clinical data (including hypertension, diabetes, coronary heart disease, and age at menarche), and laboratory parameters (such as complete blood cell count from peripheral blood). For laboratory analysis, a 3 mL sample of fasting venous blood was collected from each patient. Complete blood count parameters were subsequently measured using an automated hematology analyzer (Sysmex XN-9000). Regarding menstrual cycle information, due to the retrospective nature of the study, only menstrual status (whether the patient was menstruating at the time of blood sampling) was recorded. All patients included in this study were confirmed to be in the non-menstrual phase at the time of blood collection.

The clinical staging of endometriosis was performed using the Revised American Fertility Society (rAFS) classification system. All patients underwent laparoscopic exploration, during which the size, number, and depth of infiltration of ectopic lesions, as well as the extent and degree of pelvic adhesions, were carefully observed and recorded. Each item was scored according to the rAFS scoring sheet. The total rAFS scores were defined as follows: stage I (minimal) 1–5 points; stage II (mild) 6–15 points; stage III (moderate) 16–40 points; stage IV (severe) > 40 points.

### 2.3 Definition of endometriosis

Endometriosis is defined as the presence of endometrial glands and stroma outside the uterine cavity. In this study, the diagnosis of endometriosis was established based on laparoscopic findings combined with histopathological confirmation. During laparoscopy, characteristic lesions were identified, and excised tissue samples were subjected to pathological examination. The pathological criteria were as follows: Microscopically, typical endometriotic lesions are characterized by the presence of endometrial glands and stroma, often accompanied by fibrin and red blood cells or hemosiderin-laden macrophages. However, recurrent bleeding within ectopic endometrial implants frequently leads to the destruction and obscuration of these histological features, resulting in clinically suspected endometriosis with insufficient histological confirmation. Consequently, the identification of even sparse endometrial stromal cells or hemosiderin-laden macrophages within a lesion is considered sufficient for a definitive pathological diagnosis of endometriosis (23).

### 2.4 Definition of AISI

AISI serves as a novel biomarker that captures the balance between inflammatory activation and immune status. AISI is defined as neutrophil count × monocyte count × platelet count/lymphocyte count (all parameters measured in ×10^9^/L units).

### 2.5 External validation dataset

The external validation data for this study were derived from the National Health and Nutrition Examination Survey (NHANES), a continuous cross-sectional program designed to assess the health and nutritional status of adults and children in the United States. NHANES collects comprehensive information on demographics, socioeconomic status, dietary habits, and health-related conditions. The survey protocol was approved by the National Center for Health Statistics Ethics Review Board,^1^ and written informed consent was obtained from all participants.

For this analysis, we initially screened 31,510 participants from the NHANES 2001–2006 cycles. Participants were excluded based on the following criteria: missing data on endometriosis (*n* = 27,277), educational level (*n* = 2), marital status (*n* = 1), poverty-to-income ratio (PIR) (*n* = 190), body mass index (BMI) (n = 48), smoking or alcohol consumption status (*n* = 2), history of hypertension, diabetes, or coronary heart disease (*n* = 53), blood lipid parameters (*n* = 2,207), complete blood cell counts (*n* = 15), or age at menarche (*n* = 16). After applying these exclusions, a final sample of 1,698 participants was included in the analysis.

### 2.6 Sample size calculation

In this study, logistic regression analysis was used. For logistic regression, it is generally required that each group (i.e., each outcome category) have at least 50 samples. When the total sample size exceeds 200, bias in parameter estimation can be ignored. According to the common rule of thumb, the total sample size should be more than 100 cases and should be 5–10 times the number of predictor variables. In this study, the final number of predictor variables was 7; therefore, at least 100 cases were required (24).

### 2.7 Statistical analysis

Statistical analyses were conducted using R software (version 4.4.3) with the packages “dplyr,” “rms,” “pROC,” and “rmda.” Continuous variables were summarized as mean ± standard deviation or median (interquartile range) and compared using the *t*-test or Mann–Whitney U test, as appropriate. Categorical variables were presented as counts (percentages) and analyzed using the chi-squared test. Variables with a *p* < 0.05 in univariate analysis were entered into a multivariable logistic regression model with backward stepwise selection; only variables retaining a *p* < 0.05 were kept in the final model. Model performance was evaluated by the area under the receiver operating characteristic curve (AUC), calibration plots with 1,000 bootstrap resamples, and decision curve analysis (DCA). Odds ratios (OR) and their 95% confidence intervals (CI) were calculated, and a two-tailed *p* < 0.05 was considered statistically significant.

## 3 Results

### 3.1 Baseline characteristics

A total of 226 patients with no history of smoking, drinking, hypertension, diabetes, or coronary heart disease were included in this study, 104 (46.02%) were in the endometriosis group and 122 (53.98%) were in the non-endometriosis group. All patients in the endometriosis group were ovarian endometriosis. Among these patients, the mean age was 31.65 ± 7.21 years, the mean BMI was 21.59 ± 3.05 kg/m^2. Compared to the non-endometriosis group, the endometriosis group showed significantly higher age, total cholesterol (TC), total triglycerides (TG), low-density lipoprotein cholesterol (LDL) and AISI levels; and significantly lower BMI, lower high-density lipoprotein cholesterol (HDL) and an earlier age at menarche (all *p* < 0.05). Details were provided in Table 1.

**Table 1:** Baseline characteristics of overall patients.

Variables	Overall (*n* = 226)	Non-endometriosis (*n* = 122)	Endometriosis (*n* = 104)	*P*
Age (years)	31.65 ± 7.21	30.42 ± 7.97	33.10 ± 5.92	0.004
Height (cm)	162.56 ± 5.23	162.11 ± 5.11	163.10 ± 5.34	0.158
Weight (kg)	57.02 ± 8.33	58.00 ± 8.94	55.88 ± 7.43	0.053
BMI (kg/m^2)	21.59 ± 3.05	22.08 ± 3.28	21.01 ± 2.66	0.007
Marital status, n (%)				0.618
Unmarried	92 (40.71%)	52 (42.62%)	40 (38.46%)	
Married	134 (59.29%)	70 (57.38%)	64 (61.54%)	
Age at menarche (years)	13.31 ± 1.21	13.53 ± 1.38	13.04 ± 0.91	0.002
WBC (10^9/L)	5.69 ± 1.38	5.56 ± 1.31	5.84 ± 1.45	0.136
RBC (10^12/L)	4.37 ± 0.36	4.43 ± 0.33	4.30 ± 0.37	0.007
LYM (10^9/L)	1.72 ± 0.41	1.69 ± 0.33	1.75 ± 0.48	0.265
MON (10^9/L)	0.37 ± 0.11	0.35 ± 0.09	0.39 ± 0.12	0.002
NEU (10^9/L)	3.54 ± 1.13	3.30 ± 1.13	3.83 ± 1.07	<0.001
PLT (10^9/L)	238.41 ± 61.68	220.90 ± 52.84	258.95 ± 65.13	<0.001
AISI	189.67 ± 111.83	154.74 ± 85.22	230.64 ± 125.08	<0.001
log2-AISI	7.35 ± 0.78	7.08 ± 0.76	7.68 ± 0.68	<0.001
TC (mmol/L)	4.63 ± 0.77	4.46 ± 0.79	4.83 ± 0.69	<0.001
TG (mmol/L)	0.98 ± 0.39	0.93 ± 0.40	1.05 ± 0.38	0.024
HDL (mmol/L)	1.63 ± 0.42	1.74 ± 0.49	1.51 ± 0.27	<0.001
LDL (mmol/L)	3.01 ± 0.63	2.89 ± 0.64	3.14 ± 0.60	0.003

Continuous variables were expressed as Mean ± SD and categorical variables were expressed as NO. (%). BMI, body mass index; WBC, white blood cell count; RBC, red blood cell count; LYM, lymphocyte count; MON, monocyte count; NEU, neutrophil count; PLT, platelet count; AISI, aggregate index of systemic inflammation; TC, Total cholesterol; TG, Total triglycerides; HDL, High-density lipoprotein cholesterol; LDL, Low-density lipoprotein cholesterol.

### 3.2 Risk factors and multivariable logistic regression analysis

As shown in Table 2, univariate logistic regression analysis showed age, BMI, age at menarche, RBC, log2-AISI, TC, TG, HDL and LDL were identified as significant influencing factors endometriosis risk (all *p* < 0.05). The multivariate logistic regression results were presented in Model 1. To refine the results, backward stepwise regression was employed to develop an optimized model (Model 2). The results showed age (OR: 1.08, 95%CI: 1.02–1.14, *p* = 0.005), BMI (OR: 0.74, 95%CI: 0.64–0.85, *p* < 0.001), age at menarche (OR: 0.58, 95%CI: 0.43–0.79, *p* < 0.001), red blood cell count (RBC) (OR: 0.19, 95%CI: 0.06–0.54, *p* = 0.003), log2-AISI (OR: 4.18, 95%CI: 2.42–7.22, *p* < 0.001), TC (OR: 2.31, 95%CI: 1.40–3.82, *p* = 0.001) and HDL (OR: 0.10, 95%CI: 0.03–0.32, *p* < 0.001) were significant influencing factors.

**Table 2:** Univariate and multivariate logistic regression analysis.

Variables	Univariate analysis		Multivariate analysis			
			Model 1		Model 2	
	OR(95%CI)	*P*	OR(95%CI)	*P*	OR(95%CI)	*P*
Age (years)	1.05 (1.02, 1.10)	0.006	1.08 (1.02, 1.14)	0.005	1.08 (1.02, 1.14)	0.005
BMI (kg/m^2)	0.89 (0.81, 0.97)	0.010	0.73 (0.63, 0.85)	<0.001	0.74 (0.64, 0.85)	<0.001
Marital status, n (%)						
Unmarried	Reference					
Married	1.19 (0.70, 2.03)	0.530				
Age at menarche (years)	0.70 (0.55, 0.88)	0.003	0.57 (0.42, 0.77)	<0.001	0.58 (0.43, 0.79)	<0.001
WBC (10^9/L)	1.16 (0.96, 1.40)	0.134				
RBC (10^12/L)	0.34 (0.16, 0.75)	0.007	0.19 (0.06, 0.54)	0.002	0.19 (0.06, 0.54)	0.003
log2-AISI	3.19 (2.10, 4.85)	<0.001	4.22 (2.42, 7.34)	<0.001	4.18 (2.42, 7.22)	<0.001
TC (mmol/L)	1.99 (1.36, 2.93)	<0.001	1.83 (1.06, 3.16)	0.03	2.31 (1.40, 3.82)	0.001
TG (mmol/L)	2.18 (1.10, 4.33)	0.026	1.24 (0.46, 3.31)	0.668		
HDL (mmol/L)	0.22 (0.10, 0.47)	<0.001	0.11 (0.03, 0.38)	<0.001	0.10 (0.03, 0.32)	<0.001
LDL (mmol/L)	1.95 (1.24, 3.07)	0.004	1.66 (0.83, 3.31)	0.154		

Continuous variables were expressed as Mean ± SD and categorical variables were expressed as NO. (%). BMI, body mass index; WBC, white blood cell count; RBC, red blood cell count; LYM, lymphocyte count; MON, monocyte count; NEU, neutrophil count; PLT, platelet count; AISI, aggregate index of systemic inflammation; TC, Total cholesterol; TG, Total triglycerides; HDL, High-density lipoprotein cholesterol; LDL, Low-density lipoprotein cholesterol.

### 3.3 Development and validation of nomogram for predicting endometriosis risk

**Figure 2:**
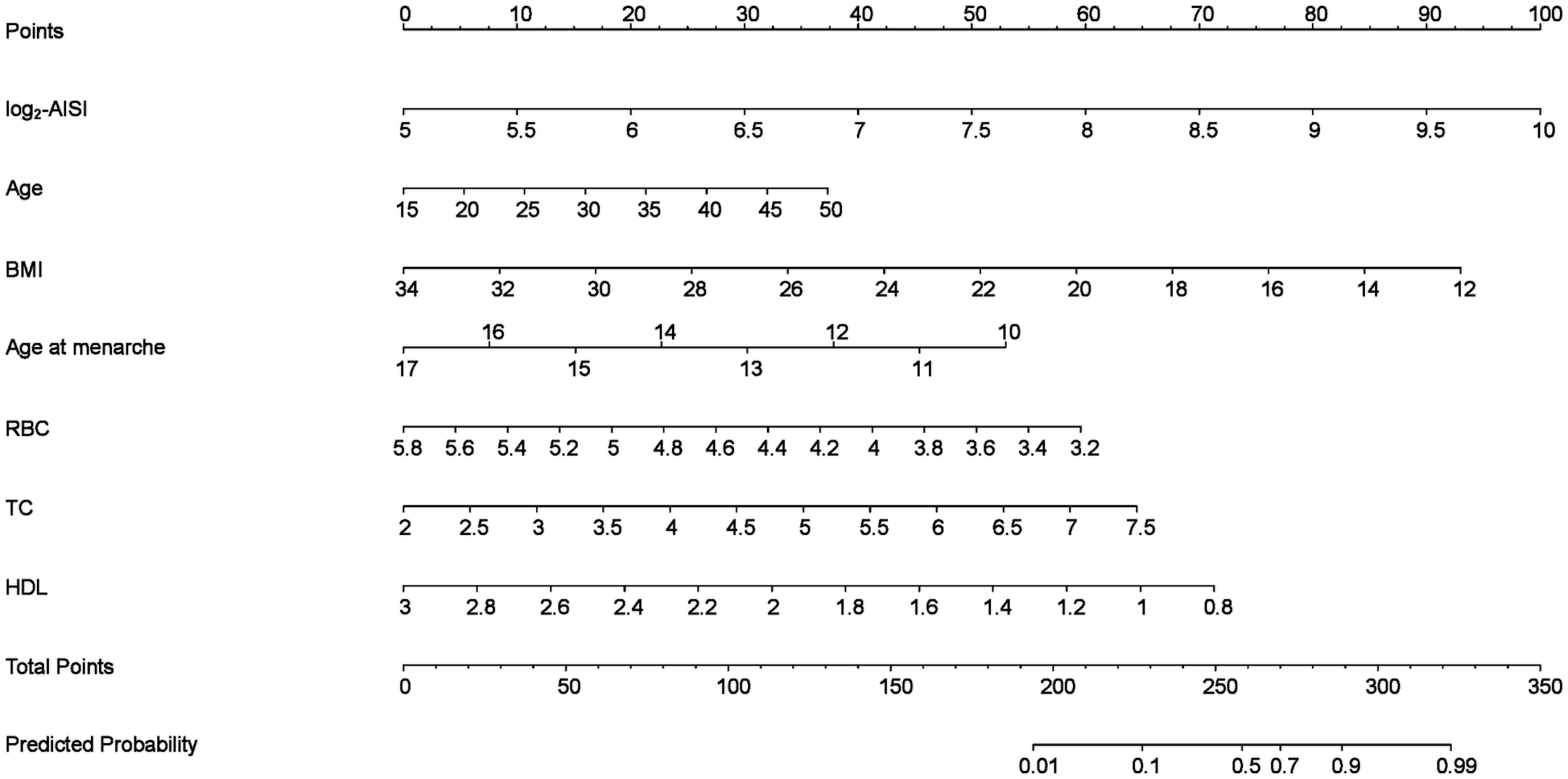
The nomogram for predicting endometriosis risk. (The points for each variable were determined based on the patient’s specific indicators and summed to yield a total score, which corresponded to a predicted probability. A higher total score indicated a greater risk of endometriosis).

Based on Model 2, we developed a nomogram by integrating factors (Figure 2). The result showed higher log2-AISI, higher age, lower BMI, earlier age at menarche, lower RBC, higher TC and lower HDL were risk factors of endometriosis. To predict endometriosis risk using the nomogram, clinicians plot the patient’s specific risk factors on the corresponding axes. The points assigned to each factor are summed to yield a total score. A higher total score indicates a greater risk of endometriosis.

**Figure 3:**
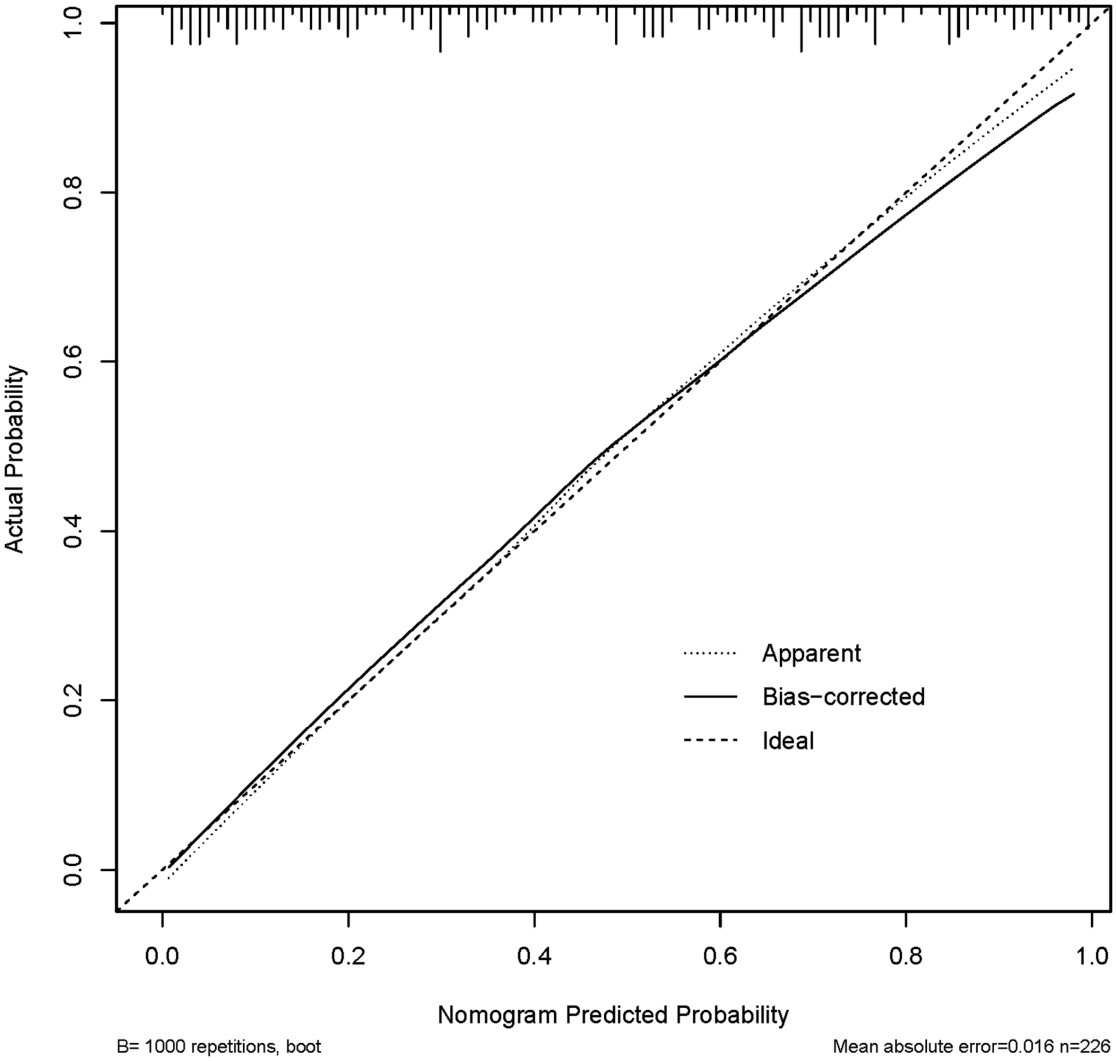
The calibration curve of the nomogram for endometriosis risk. (The calibration curve was generated with 1,000 bootstrap repetitions. In the plot, the diagonal dashed line denotes a perfect prediction, and the solid line represents the observed model performance. Closer agreement between the two lines signifies better predictive accuracy).

Calibration curve was used to evaluate the reliability of the Nomogram model’s predicted probabilities. Using the bootstrap method (1,000 repetitions), a calibration curve was plotted. In the plot, the dashed diagonal line indicates ideal prediction, and the solid line reflects the observed model performance. The predictive accuracy is considered better when these two lines are in closer proximity. As shown in Figure 3, the absolute error between the simulated and actual curves was 0.016, indicating good calibration of the model.

**Figure 4:**
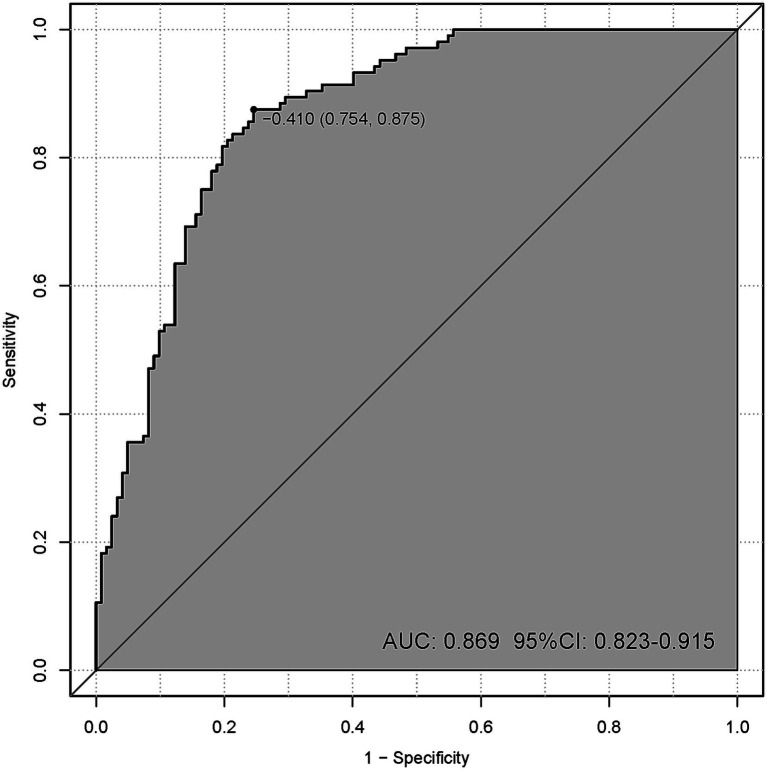
ROC curve of the nomogram for endometriosis risk. (An AUC > 0.7 indicates that the model demonstrates relatively good discriminatory ability).

The nomogram model achieved an AUC of 0.869 (95% CI: 0.823–0.915), indicating strong discriminatory ability (Figure 4). The optimal cutoff value was −0.410, corresponding to a maximum Youden’s index of 0.629, with a predictive sensitivity of 75.4% and a specificity of 87.5%. In comparison, the model excluding log2-AISI (Supplementary Figure S1) achieved an AUC of 0.811 (95% CI: 0.757–0.866), whereas the model containing only log2-AISI (Supplementary Figure S2) yielded an AUC of 0.721 (95% CI: 0.656–0.786).

**Figure 5:**
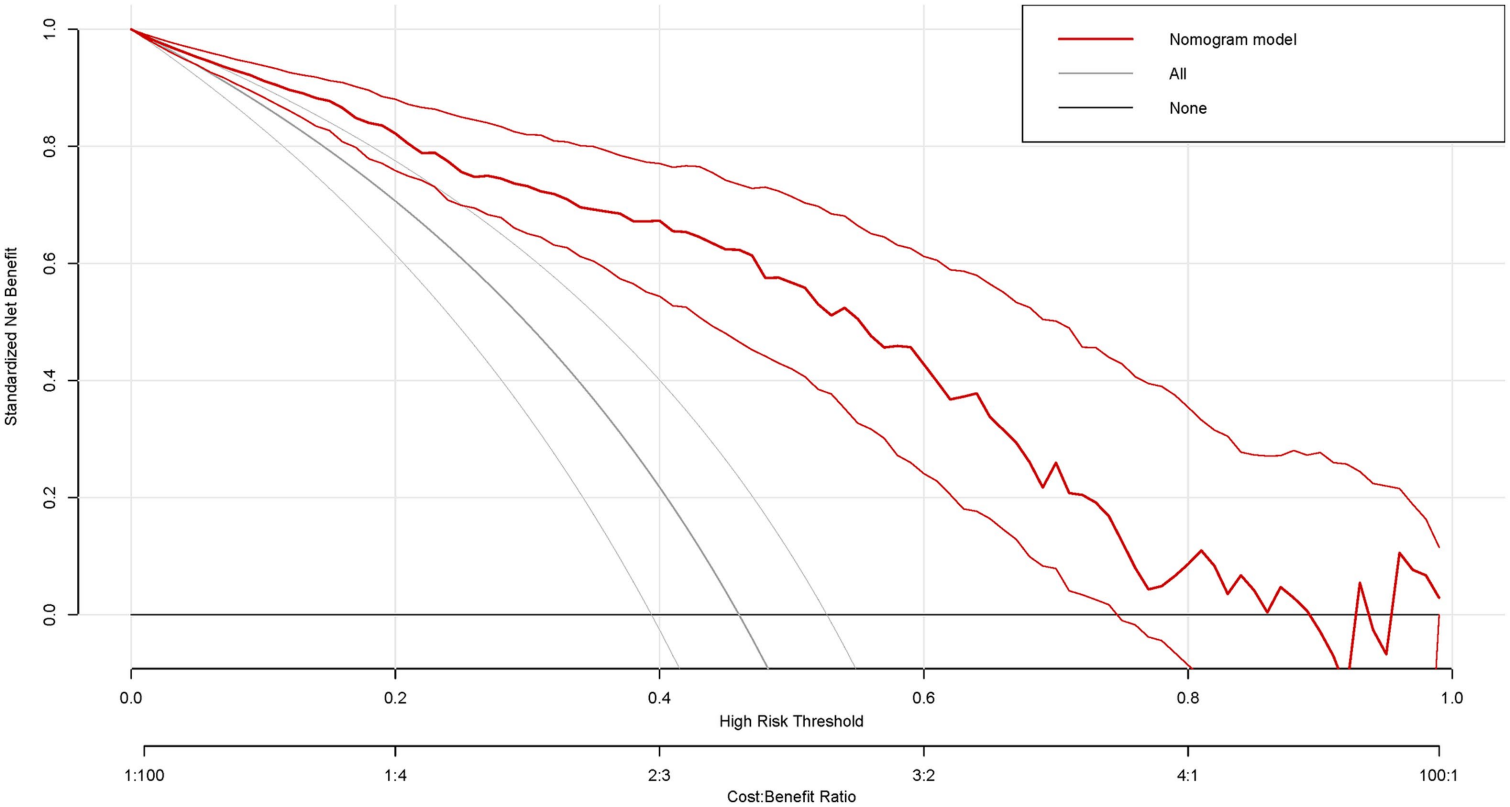
The decision curve of the nomogram for endometriosis risk. (Across a threshold probability range of 10–90%, the nomogram provided a greater net benefit than both the “treat-all” and “treat-none” strategies, thereby demonstrating its clinical utility in routine practice).

DCA was performed for the nomogram model to evaluate their net clinical benefit across a spectrum of threshold probabilities (Figure 5). Within the clinically relevant range (approximately 10–90%), the nomogram model demonstrated a standardized net benefit that exceeded both the “treat-all” and “treat-none” strategies, underscoring their substantial clinical utility.

### 3.4 External validation

The external validation data were derived from the NHANES (2001–2006). Participants had a mean age of 35.70 ± 10.01 years and a mean BMI of 28.87 ± 7.25 kg/m^2. Inflammatory indices (MON, NEU, PLT, AISI) were significantly higher in the endometriosis group compared to the non-endometriosis group (all *p* < 0.05). Additionally, the log2-AISI was significantly higher in the endometriosis group than in the non-endometriosis group (8.75 ± 0.87 vs. 8.11 ± 0.96, *p* < 0.001). Although statistically non-significant, the results suggested higher levels of TC, TG, and LDL, along with a lower level of HDL, in the endometriosis group compared with the non-endometriosis group. Details were shown in Supplementary Table S1.

Univariate (OR: 2.16, 95%CI: 1.77–2.65, *p* < 0.001) and multivariate logistic regression analyses (OR: 2.39, 95%CI: 1.92–2.98, *p* < 0.001) both demonstrated that log2-AISI was identified as a risk factor for the development of endometriosis (Supplementary Table S2).

### 3.5 Relationship between AISI and endometriosis risk

Based on the logistic regression analyses presented in Table 3, a significant positive association was observed between log2-AISI and endometriosis risk in both datasets. In the institutional data, the unadjusted analysis (Model 1) revealed a significant association (OR: 3.19, 95% CI: 2.10–4.85, *p* < 0.001). This association persisted and strengthened after adjusting for age and BMI in Model 2 (OR: 3.58, 95% CI: 2.29–5.60, *p* < 0.001). Further adjustment for all covariates in Model 3 yielded similar results (OR: 3.77, 95% CI: 2.23–6.37, *p* < 0.001). A consistent positive relationship was also identified in the NHANES data. The crude model (Model 1) showed a significant association (OR: 2.16, 95% CI: 1.77–2.65, *p* < 0.001). Adjustment for age and BMI in Model 2, this positive association remained (OR: 2.43, 95% CI: 1.96–3.01, *p* < 0.001). In the fully adjusted Model 3, the association remained statistically significant (OR: 2.38, 95% CI: 1.90–2.98, *p* < 0.001).

**Table 3:** Relationship between AISI and endometriosis risk using logistic regression analyses.

Data types	Variables	Model 1		Model 2		Model 3	
		OR(95% CI)	*P*	OR(95% CI)	*P*	OR(95% CI)	*P*
Institutional data	log2-AISI	3.19 (2.10 ~ 4.85)	<0.001	3.58 (2.29 ~ 5.60)	<0.001	3.77 (2.23 ~ 6.37)	<0.001
NHANES data	log2-AISI	2.16 (1.77 ~ 2.65)	<0.001	2.43 (1.96 ~ 3.01)	<0.001	2.38 (1.90 ~ 2.98)	<0.001

OR, odds ratio; CI, Confidence interval. Model 1: No covariates adjusted. Model 2: Adjusted for age, BMI. Model 3: The institutional data were adjusted for age, BMI, marital status, age at menarche, WBC, RBC, TC, TG, HDL and LDL; NHANES data were adjusted for age, BMI, race, family PIR, education, marital status, alcohol user, smoking, hypertension, diabetes, coronary heart disease, age at menarche, TC, TG, HDL and LDL.

### 3.6 Relationship between AISI and endometriosis stages

According to the Revised American Fertility Society (rAFS) classification, among the 104 patients with endometriosis, 45 (43.27%) were III stage and 59 (56.73%) were IV stage, with no patients in stages I or II. Compared to the III stage group, the IV stage group showed significantly higher log2-AISI (7.96 ± 0.65 vs. 7.32 ± 0.52, *p* < 0.001). Details were shown in Supplementary Table S2.

**Figure 6:**
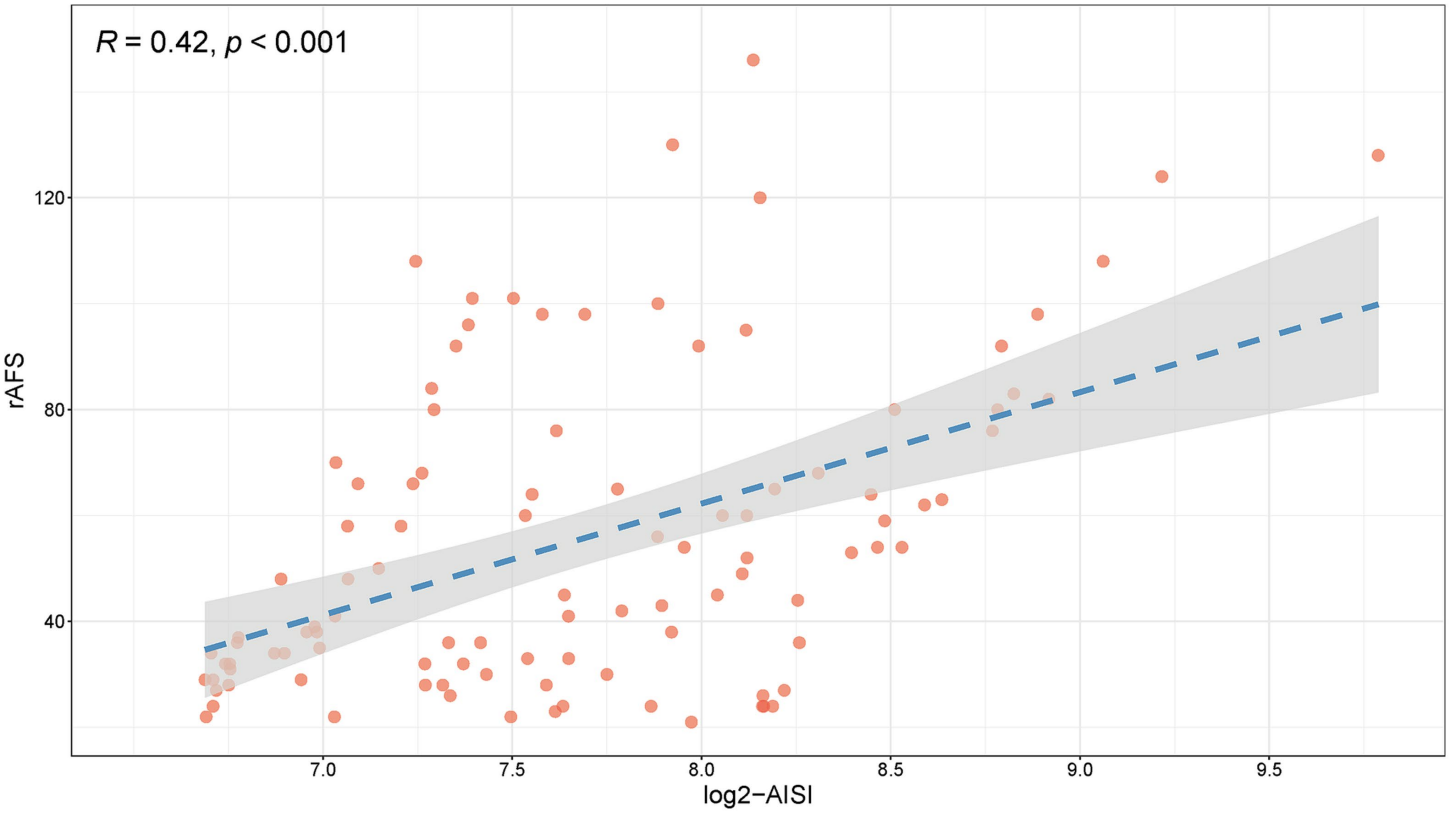
Relationship between log2-AISI and rAFS score. (An *R* value > 0 with a *p* < 0.05, indicating a positive correlation).

To further investigate the correlation between log2-AISI and endometriosis stages, Spearman’s analysis was performed to evaluate the association between log2-AISI and rAFS scores. The results showed a significant positive correlation between log2-AISI (Figure 6) (*R* = 0.42, *p* < 0.001) and the rAFS score.

## 4 Discussion

Currently, the diagnosis of endometriosis relies primarily on clinical presentation, imaging findings, and ultimately laparoscopic visualization with histological confirmation, with no widely accepted non-invasive biomarkers available for routine clinical use. Furthermore, many women are reluctant to undergo this invasive procedure, resulting in delayed diagnosis in a substantial number of patients (25). Transvaginal ultrasound and MRI demonstrate moderate diagnostic accuracy but are operator-dependent and resource-intensive (26). Our study demonstrated that log2-AISI was independently associated with endometriosis risk in both the institutional cohort and the NHANES external validation dataset.

The association between AISI and endometriosis risk is biologically plausible and likely reflects the multifaceted role of systemic inflammation in endometriosis pathogenesis. As a composite index integrating neutrophils, platelets, monocytes, and lymphocytes, AISI captures the complex interplay among these cellular components that collectively contribute to the establishment and progression of endometriotic lesions. Notably, the individual components of AISI have been linked to specific inflammatory mediators previously implicated in endometriosis. For instance, matrix metalloproteinase-9 (MMP-9), primarily derived from neutrophils and macrophages, has been shown to be elevated in peritoneal fluid and ectopic lesions of endometriosis patients, where it facilitates extracellular matrix degradation and promotes lesion invasion and angiogenesis (27, 28). Neutrophil gelatinase-associated lipocalin (NGAL), often complexed with MMP-9, enhances MMP-9 activity and has been proposed as a potential biomarker for endometriosis severity (29). Additionally, reactive oxygen species such as hydrogen peroxide (H₂O₂), released by activated neutrophils and macrophages, contribute to oxidative stress within the peritoneal microenvironment, promoting DNA damage, cellular proliferation, and survival of ectopic endometrial cells (30, 31). These pro-inflammatory and pro-oxidant mediators are primarily derived from the cellular constituents of AISI, particularly neutrophils and monocytes. Neutrophils, as the most abundant circulating leukocytes, represent the first line of defense in acute inflammatory responses. In the context of endometriosis, neutrophils have been shown to infiltrate peritoneal fluid and ectopic lesions, where they contribute to tissue damage and inflammation through the release of reactive oxygen species, matrix metalloproteinases, and neutrophil extracellular traps (NETs) (32, 33). NETs, in particular, have been implicated in promoting adhesion and proliferation of endometrial cells at ectopic sites, as well as in activating peritoneal mesothelial cells to create a permissive microenvironment for lesion implantation (34). The elevated neutrophil counts reflected in higher AISI values may indicate enhanced neutrophil activation and NETosis, thereby facilitating lesion establishment. Platelets have emerged as critical contributors to endometriosis pathogenesis beyond their traditional hemostatic functions. Within endometriotic lesions, platelets have been observed to undergo activation and aggregation, releasing growth factors such as transforming growth factor-beta (TGF-β), vascular endothelial growth factor (VEGF) and platelet-derived growth factor (PDGF) that promote angiogenesis, cell proliferation, and tissue remodeling (35, 36). Notably, repeated bleeding within endometriotic lesions leads to iron overload and oxidative stress, which further amplifies platelet activation and creates a self-perpetuating cycle of inflammation and tissue injury (37). Platelet-derived TGF-β has been specifically implicated in inducing epithelial-mesenchymal transition (EMT) in endometrial epithelial cells, a process thought to be critical for lesion invasiveness and fibrosis (38). The inclusion of platelets in AISI therefore captures this pro-inflammatory and pro-fibrotic axis that contributes to disease progression. Monocytes and their differentiated progeny, macrophages, occupy a central position in endometriosis-associated inflammation. Upon recruitment to the peritoneal cavity and ectopic lesions, monocytes differentiate into macrophages that exhibit predominantly M2-polarized phenotypes in established disease (39, 40). While M2 macrophages are classically associated with tissue repair and anti-inflammatory functions, in the endometriosis microenvironment they paradoxically promote lesion survival through secretion of IL-10, TGF-β, and VEGF, which suppress cytotoxic immune responses while supporting angiogenesis and lesion growth (41–43). Furthermore, endometriosis-associated macrophages have been shown to exhibit impaired phagocytic capacity, leading to inefficient clearance of apoptotic cells and erythrocytes, thereby perpetuating inflammation through accumulation of iron and cellular debris (44). The monocyte component of AISI may therefore reflect the expansion of this pro-lesional macrophage lineage. Lymphocytes, particularly cytotoxic CD8^+^ T cells and natural killer (NK) cells, constitute the primary anti-endometriotic immune defense through their capacity to recognize and eliminate ectopic endometrial cells (45, 46). Accumulating evidence indicates that women with endometriosis exhibit impaired cytotoxic activity of both peripheral and peritoneal NK cells, as well as functional alterations in T cell subsets characterized by reduced cytotoxicity and increased regulatory T cell (Treg) proportions (46, 47). This lymphocyte dysfunction creates an immunosuppressive environment that permits immune evasion of ectopic endometrial implants.

Beyond the AISI-associated inflammatory mechanisms, our study identified several additional factors independently associated with endometriosis risk that merit discussion. Age demonstrated a positive association with endometriosis risk, consistent with the cumulative exposure to retrograde menstruation and the progressive nature of the disease (48). BMI showed an inverse association, aligning with previous observations that lower BMI confers increased endometriosis risk, possibly due to altered estrogen metabolism and reduced adipose tissue-derived hormone levels (49, 50). Earlier age at menarche emerged as a significant risk factor, reflecting prolonged cumulative exposure to cyclic menstruation and estrogen stimulation (51). Elevated TC and reduced HDL are particularly noteworthy and suggest potential metabolic-inflammatory interactions in endometriosis pathogenesis. Dyslipidemia has been increasingly recognized in endometriosis patients, with proposed mechanisms including estrogen-mediated effects on lipid metabolism, chronic inflammation-induced alterations in hepatic lipoprotein synthesis, and shared genetic or environmental determinants (52, 53). Oxidized LDL particles can activate Toll-like receptor 4 signaling on macrophages and endometrial cells, promoting pro-inflammatory cytokine production and creating a permissive environment for lesion establishment (54). Conversely, HDL possesses anti-inflammatory and antioxidant properties, and its reduction may compromise these protective functions (55). The incorporation of lipid parameters alongside AISI in our nomogram therefore captures complementary aspects of the metabolic-inflammatory axis in endometriosis.

External validation using the NHANES dataset substantially strengthens our findings by demonstrating the generalizability of the AISI-endometriosis association across different populations and healthcare settings. In addition, compared to established biomarkers, the combination of systemic immune-inflammation index (SII), systemic inflammation response index (SIRI), neutrophil-to-lymphocyte ratio (NLR) and pan-immune-inflammation value (PIV) yielded an AUC of 0.796 (56), while the Imaging features achieved an AUC of 0.744 (57). In contrast, the predictive model established in this study demonstrated superior discriminatory performance, with an AUC of 0.896. Furthermore, AISI alone achieved an AUC of 0.721, highlighting its significant diagnostic value in endometriosis.

Several limitations of this study should be acknowledged. First, the retrospective design precludes establishment of causal relationships between AISI and endometriosis; while we observed a strong and consistent association, prospective cohort studies are needed to clarify the temporal sequence and determine whether elevated AISI precedes disease onset or reflects existing disease-related inflammation. Second, the institutional cohort, while well-characterized with pathological confirmation, had a relatively modest sample size (*n* = 226) and was derived from a single center, potentially limiting generalizability. Although external validation using NHANES partially addresses this concern, the NHANES endometriosis diagnosis relied on self-report rather than pathological confirmation, introducing potential misclassification bias. Third, despite adjustment for multiple confounders, residual confounding from unmeasured variables, including detailed hormonal profiles, specific inflammatory cytokines (IL-6, TNF-*α*, CRP), and genetic factors. Fourth, the cross-sectional nature of both datasets prevented assessment of AISI dynamics over time and their relationship with disease progression or treatment response. Fifth, the study population was restricted to non-smoking, non-drinking individuals without major comorbidities to minimize confounding, which may limit applicability to broader, more heterogeneous populations. Sixth, we were unable to obtain the exact day of the menstrual cycle for female participants. Hormonal fluctuations during the menstrual cycle may affect systemic inflammatory markers. Based on these limitations, several directions for future research are warranted. First, large-scale prospective cohort studies with serial AISI measurements are needed to establish the temporal relationship between systemic inflammation and endometriosis development, and to determine whether AISI can predict incident disease among asymptomatic high-risk populations. Second, multicenter studies across diverse geographic and ethnic populations should validate our findings and establish optimal AISI cutoff values for clinical risk stratification. Third, integration of AISI with more specific diagnostic modalities, such as transvaginal ultrasound features, MRI findings, or novel proteomic biomarkers, may further enhance predictive accuracy and facilitate development of multi-modal risk assessment tools. Fourth, mechanistic studies exploring the precise biological pathways linking AISI components to endometriosis pathogenesis. Fifth, longitudinal studies examining AISI changes in response to medical or surgical interventions could establish its utility as a monitoring biomarker for treatment efficacy and disease recurrence. Finally, exploration of AISI in specific endometriosis subtypes may reveal differential associations that could inform personalized diagnostic and therapeutic approaches.

## 5 Conclusion

In conclusion, this study demonstrated that AIS was independently and positively associated with endometriosis risk. The nomogram incorporating AISI with age, BMI, age at menarche, RBC, TC, and HDL provides a readily accessible, cost-effective tool for early risk stratification that could complement existing diagnostic pathways and potentially reduce diagnostic delays. External validation using NHANES data supports the generalizability of these findings. While further prospective and mechanistic studies are warranted, our results position AISI as a promising adjunctive biomarker for endometriosis risk assessment and highlight the central role of systemic inflammation.

## Data Availability

The original contributions presented in the study are included in the article/Supplementary material, further inquiries can be directed to the corresponding author.

## Footnotes

1
https://www.cdc.gov/nchs/nhanes/about/erb.html
