# Supplementary material for: Associations between aggregate index of systemic inflammation and endometriosis risk utilizing logistic regression analysis
Source: Front Med (Lausanne). 2026 May 4;13:1817928. doi: 10.3389/fmed.2026.1817928 (PMC13180812; doi:10.3389/fmed.2026.1817928)
Supplement: Supplementary file 1 [file Supplementary_file_1.docx]

**Table S1 Baseline characteristics of participants from NHANES 2001-2006**

| Variables | Overall (n=1698) | Non-endometriosis (n=122) | Endometriosis (n=104) | P |
| --- | --- | --- | --- | --- |
| Age (years) | 35.70 ± 10.01 | 35.28 ± 10.04 | 40.35 ± 8.38 | <0.001 |
| BMI (kg/m^2^) | 28.87 ± 7.25 | 28.94 ± 7.35 | 28.19 ± 5.92 | 0.161 |
| Race, n (%) |  |  |  | <0.001 |
| Mexican American | 366 (21.55%) | 358 (22.99%) | 8 (5.67%) |  |
| Other Hispanic | 58 (3.42%) | 53 (3.40%) | 5 (3.55%) |  |
| Non-Hispanic White | 834 (49.12%) | 740 (47.53%) | 94 (66.67%) |  |
| Non-Hispanic Black | 359 (21.14%) | 329 (21.13%) | 30 (21.28%) |  |
| Other race, including multi-racial | 81 (4.77%) | 77 (4.95%) | 4 (2.84%) |  |
| Family PIR | 2.69 ± 1.65 | 2.66 ± 1.64 | 3.03 ± 1.67 | 0.011 |
| Education, n (%) |  |  |  | 0.002 |
| Less than high school | 375 (22.08%) | 359 (23.06%) | 16 (11.35%) |  |
| High school and above | 1323 (77.92%) | 1198 (76.94%) | 125 (88.65%) |  |
| Marital status, n (%) |  |  |  | 0.901 |
| Living alone | 540 (31.80%) | 494 (31.73%) | 46 (32.62%) |  |
| Married/Living with partner | 1158 (68.20%) | 1063 (68.27%) | 95 (67.38%) |  |
| Alcohol user, n (%) |  |  |  | 0.042 |
| No | 647 (38.10%) | 605 (38.86%) | 42 (29.79%) |  |
| Yes | 1051 (61.90%) | 952 (61.14%) | 99 (70.21%) |  |
| Smoking, n (%) |  |  |  | 0.002 |
| No | 1047 (61.66%) | 978 (62.81%) | 69 (48.94%) |  |
| Yes | 651 (38.34%) | 579 (37.19%) | 72 (51.06%) |  |
| Hypertension, n (%) |  |  |  | 0.002 |
| No | 1379 (81.21%) | 1279 (82.15%) | 100 (70.92%) |  |
| Yes | 319 (18.79%) | 278 (17.85%) | 41 (29.08%) |  |
| Diabetes, n (%) |  |  |  | 0.808 |
| No | 1625 (95.70%) | 1489 (95.63%) | 136 (96.45%) |  |
| Yes | 73 (4.30%) | 68 (4.37%) | 5 (3.55%) |  |
| Coronary heart disease, n (%) |  |  |  | 0.581 |
| No | 1688 (99.41%) | 1548 (99.42%) | 140 (99.29%) |  |
| Yes | 10 (0.59%) | 9 (0.58%) | 1 (0.71%) |  |
| Age at menarche (years) | 12.52 ± 1.66 | 12.58 ± 1.67 | 11.90 ± 1.36 | <0.001 |
| TG (mg/dL) | 123.37 ± 69.93 | 122.55 ± 69.46 | 132.34 ± 74.53 | 0.135 |
| TC (mg/dL) | 197.05 ± 42.72 | 196.51 ± 42.86 | 202.95 ± 40.82 | 0.076 |
| LDL (mg/dL) | 113.11 ± 35.61 | 112.71 ± 35.64 | 117.55 ± 35.15 | 0.119 |
| HDL (mg/dL) | 59.26 ± 16.25 | 59.29 ± 16.18 | 58.94 ± 17.00 | 0.815 |
| LYM | 2.00 ± 0.61 | 2.00 ± 0.62 | 2.03 ± 0.60 | 0.519 |
| MON | 0.51 ± 0.17 | 0.49 ± 0.15 | 0.67 ± 0.25 | <0.001 |
| NEU | 4.47 ± 1.79 | 4.44 ± 1.78 | 4.88 ± 1.78 | 0.005 |
| PLT | 284.67 ± 73.36 | 283.60 ± 73.83 | 296.56 ± 67.05 | 0.031 |
| AISI | 354.42 ± 245.19 | 339.20 ± 223.03 | 522.49 ± 380.62 | <0.001 |
| log2-AISI | 8.16 ± 0.97 | 8.11 ± 0.96 | 8.75 ± 0.87 | <0.001 |

BMI: Body mass index;PIR: Poverty income ratio; TC: Total cholesterol;TG: Total triglycerides; HDL: High-density lipoprotein cholesterol; LDL: Low-density lipoprotein cholesterol; LYM: lymphocyte count; MON: monocyte count; NEU: neutrophil count; PLT: platelet count; AISI: aggregate index of systemic inflammation.

**Table S2 Baseline characteristics of endometriosis**

| Variables | Overall (n=104) | III stage (n=45) | IV stage (n=59) | P |
| --- | --- | --- | --- | --- |
| Age (years) | 33.10 ± 5.92 | 32.11 ± 5.59 | 33.85 ± 6.09 | 0.134 |
| Height (cm) | 163.10 ± 5.34 | 164.31 ± 5.44 | 162.17 ± 5.11 | 0.044 |
| Weight (kg) | 55.88 ± 7.43 | 55.02 ± 8.31 | 56.54 ± 6.68 | 0.319 |
| BMI (kg/m^2) | 21.01 ± 2.66 | 20.38 ± 3.03 | 21.49 ± 2.25 | 0.044 |
| Marital Status, n (%) |  |  |  | 0.194 |
| Unmarried | 40 (38.46%) | 21 (46.67%) | 19 (32.20%) |  |
| Married | 64 (61.54%) | 24 (53.33%) | 40 (67.80%) |  |
| Age at menarche (years) | 13.04 ± 0.91 | 12.80 ± 0.97 | 13.22 ± 0.83 | 0.022 |
| rAFS | 55.56 ± 29.50 | 30.18 ± 5.54 | 74.92 ± 25.32 | <0.001 |
| WBC (10^9/L) | 5.84 ± 1.45 | 5.56 ± 1.22 | 6.05 ± 1.59 | 0.076 |
| RBC (10^12/L) | 4.30 ± 0.37 | 4.29 ± 0.39 | 4.31 ± 0.36 | 0.781 |
| LYM (10^9/L) | 1.75 ± 0.48 | 1.80 ± 0.44 | 1.72 ± 0.51 | 0.407 |
| MON (10^9/L) | 0.39 ± 0.12 | 0.36 ± 0.10 | 0.42 ± 0.13 | 0.018 |
| NEU (10^9/L) | 3.83 ± 1.07 | 3.60 ± 0.87 | 4.00 ± 1.18 | 0.049 |
| PLT (10^9/L) | 258.95 ± 65.13 | 233.51 ± 54.83 | 278.36 ± 66.09 | <0.001 |
| AISI | 230.64 ± 125.08 | 170.20 ± 64.46 | 276.74 ± 140.13 | <0.001 |
| log2-AISI | 7.68 ± 0.68 | 7.32 ± 0.52 | 7.96 ± 0.65 | <0.001 |
| TC (mmol/L) | 4.83 ± 0.69 | 4.84 ± 0.80 | 4.82 ± 0.59 | 0.894 |
| TG (mmol/L) | 1.05 ± 0.38 | 1.00 ± 0.39 | 1.09 ± 0.37 | 0.244 |
| HDL (mmol/L) | 1.51 ± 0.27 | 1.53 ± 0.24 | 1.49 ± 0.29 | 0.502 |
| LDL (mmol/L) | 3.14 ± 0.60 | 3.16 ± 0.73 | 3.13 ± 0.49 | 0.792 |

Continuous variables were expressed as Mean ± SD and categorical variables were expressed as NO. (%). BMI: body mass index; rAFS: revised american fertility society; WBC: white blood cell count; RBC: red blood cell count; LYM: lymphocyte count; MON: monocyte count; NEU: neutrophil count; PLT: platelet count; AISI: aggregate index of systemic inflammation; TC: Total cholesterol;TG: Total triglycerides; HDL: High-density lipoprotein cholesterol; LDL: Low-density lipoprotein cholesterol.
