# Supplementary material for: Associations between aggregate index of systemic inflammation and endometriosis risk utilizing logistic regression analysis
Source: Front Med (Lausanne). 2026 May 4;13:1817928. doi: 10.3389/fmed.2026.1817928 (PMC13180812; doi:10.3389/fmed.2026.1817928)
Supplement: Supplementary file 2 [file Supplementary_file_2.docx]

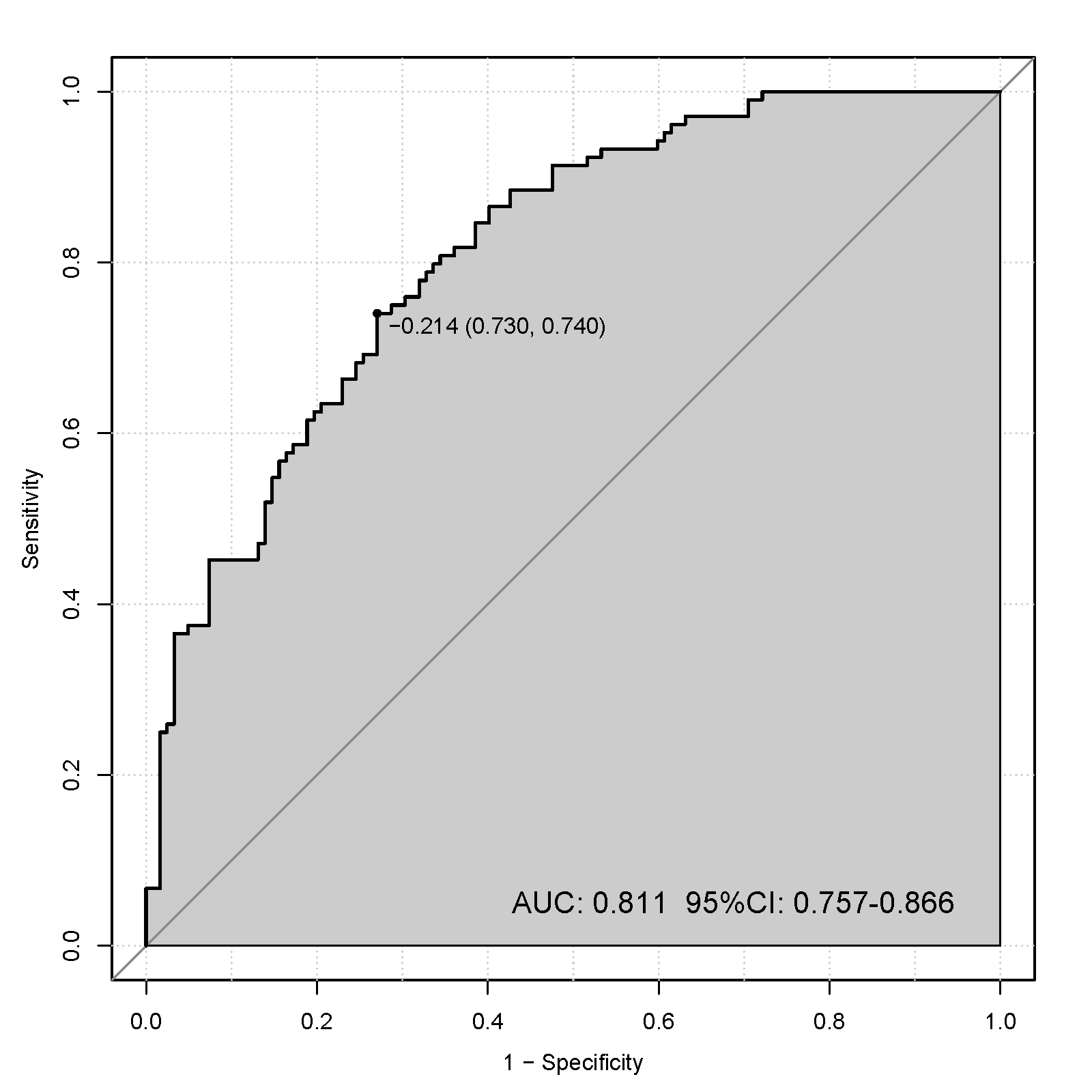


Figure S1. ROC curve of the nomogram without log2-AISI.


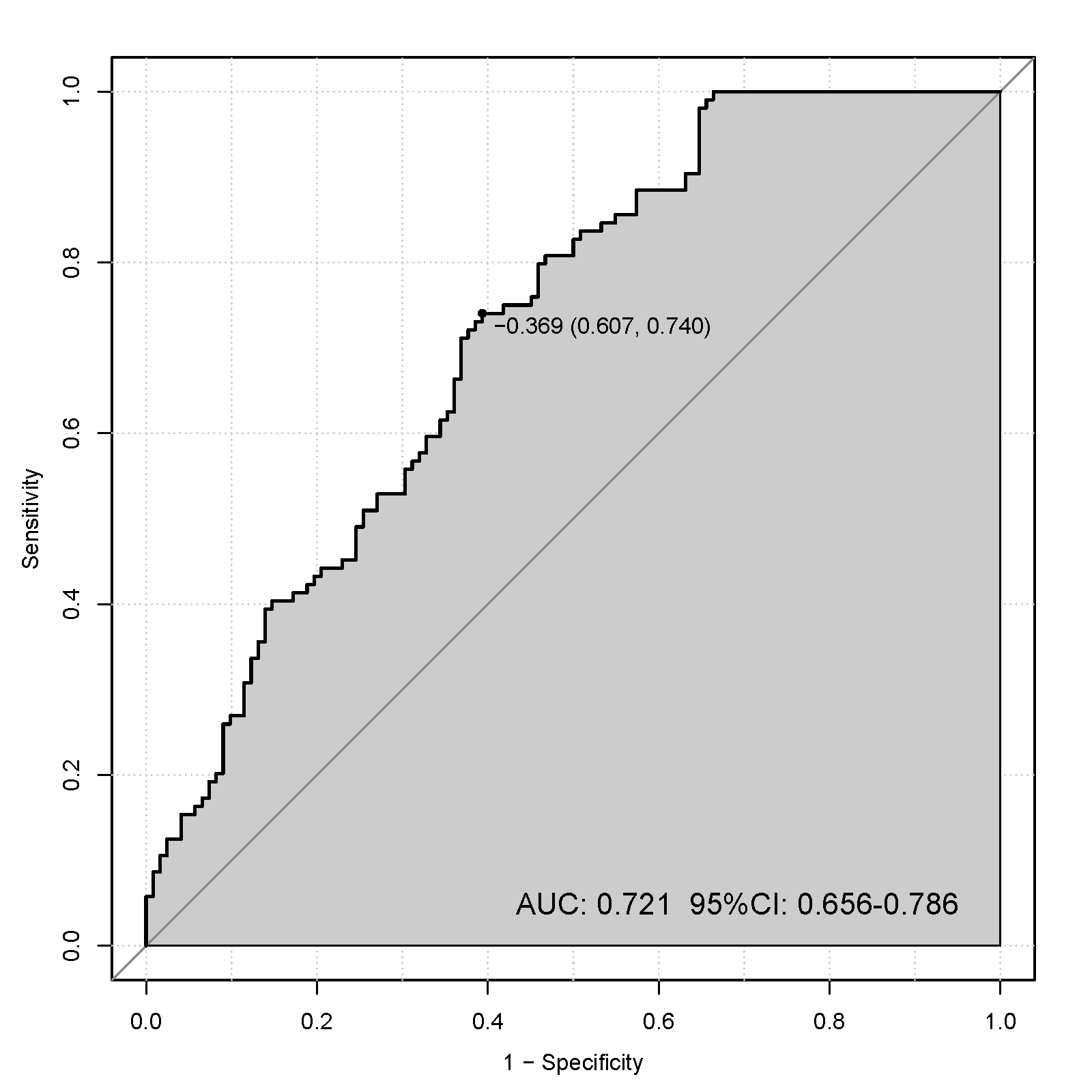


Figure S2. ROC curve of the nomogram with only log2-AISI.
